# Letter to the editor regarding: “cystoid macular edema secondary to gyrate atrophy in a child treated with sub-tenon injection of triamcinolone acetonide”

**Published:** 2018

**Authors:** Ayman G Elnahry

**Affiliations:** * Department of Ophthalmology, Faculty of Medicine, Cairo University, Cairo, Egypt

## Dear Editor,

I read with great interest a recent case report by Alparslan et al. published in your journal regarding a case of cystoid macular edema (CME) associated with gyrate atrophy (GA) of the choroid and retina in a child, which partially improved with a subtenon injection of triamcinolone acetonide [**1**]. Recently, we have also published a case report of CME, or better termed intraretinal cystic spaces, associated with GA in a child, also unable to undergo dietary modifications, that partially improved with intravitreal bevacizumab injections [**2**]. This was the first report describing the use of bevacizumab to treat this condition. The pathogenesis of CME associated with GA is still unknown, however, new data from optical coherence tomography angiography suggest that retinal ischemia and vascular alterations may play a role, and indicate that anti vascular endothelial growth factor agents, such as bevacizumab, could be useful [**2**,**3**]. Previously, intravitreal triamcinolone acetonide has been shown to improve CME associated with GA [**4**]. Both intravitreal and subtenon triamcinolone injections, however, are associated with an increased risk of intraocular pressure elevation and cataract formation especially with the repeated injections that may be required in this chronic condition. This could have negative consequences since most patients with gyrate atrophy are already at an increased risk of early cataract formation and early surgery would result in loss of accommodation among other possible surgical complications [**5**]. Bevacizumab is devoid of these complications and could therefore be a potentially safe and effective alternative for the treatment of this condition.

**Financial support**

None.

**Conflicts of Interest and Source of Funding**

None.
